# Complement activation products in the circulation and urine of primary membranous nephropathy

**DOI:** 10.1186/s12882-019-1509-5

**Published:** 2019-08-09

**Authors:** Mu-fan Zhang, Jing Huang, Yi-miao Zhang, Zhen Qu, Xin Wang, Fang Wang, Li-qiang Meng, Xu-yang Cheng, Zhao Cui, Gang Liu, Ming-hui Zhao

**Affiliations:** 1 Department of Medicine, Renal Division, Peking University First Hospital, Beijing, 100034 China; 2 Institute of Nephrology, Peking University, Beijing, 100034 China; 3 Key Laboratory of Renal Disease, Ministry of Health of China, Beijing, 100034 China; 4 Key Laboratory of CKD Prevention and Treatment, Ministry of Education of China, Beijing, 100034 China; 5 Peking-Tsinghua Center for Life Sciences, Beijing, China

**Keywords:** Primary membranous nephropathy, Complement, C3a, C5a, PLA2R

## Abstract

**Background:**

Complement activation plays a substantial role in the pathogenesis of primary membranous nephropathy (pMN). C5b-9, C3c, MBL, and factor B have been documented in the subepithelial immune deposits. However, the changing of complement activation products in circulation and urine is not clear.

**Methods:**

We measured the circulating and urinary levels of C1q, MBL, C4d, Bb, properdin, C3a, C5a, and sC5b-9, in 134 patients with biopsy-proven pMN, by enzyme-linked immunosorbent assay. All the plasma values were corrected by eGFR and all the urinary values were corrected by urinary creatinine and urinary protein excretion. Anti-PLA2R antibodies were measured in all patients.

**Results:**

The plasma complement activation products were elevated both in the patients with and without anti-PLA2R antibodies. C3a levels were remarkably increased in the circulation and urine, much higher than the elevated levels of C5a. C5b-9 was in normal range in plasma, but significantly higher in urine. The urinary C5a had a positive correlation with anti-PLA2R antibody levels and urinary protein. The plasma level of C4d was elevated, but C1q and MBL were comparable to healthy controls. Positive correlations were observed between plasma C4d/MBL and urinary protein, only in the patients with positive anti-PLA2R antibodies but not in those without. The plasma level of Bb was elevated and had positive correlation with urinary protein only in the patients without anti-PLA2R antibodies.

**Conclusion:**

Complement activation products were remarkable increased in pMN and may serve as sensitive biomarkers of disease activity. The complement may be activated through lectin pathway with the existence of anti-PLA2R antibodies, while through alternative pathway in the absence of antibody.

## Background

Primary membranous nephropathy (pMN), an autoimmune-mediated glomerular disease, is one of the most common causes of nephrotic syndrome in adults and might progress to end-stage renal disease (ESRD). The renal pathology of MN is characterized by the deposition of IgG and complements [1–3]. Serum IgG4 specific for the M-type phospholipase A2 receptor (PLA2R), a transmembrane glycoprotein expressed on the glomerular podocyte, is elevated in 70–80% of patients with pMN and is believed to be the pathogenic antibody [1].

Although IgG4 does not efficiently activate complement through the classical pathway, deposition of C4d is detectable in essentially 100% of pMN patients [4, 5]. Previous studies have demonstrated the among the subepithelial deposits in pMN, mannose binding lectin (MBL) and hypogalactosylated IgG were important components, and the latter binds with MBL in order to trigger the lectin pathway and thus complement cascades [6]. These data suggest that MBL-initiated complement activation is pathogenic in pMN. However, a recent report suggested that pMN can develop in patients with complete MBL deficiency due to sequence polymorphisms in the promoter and the coding region [7]. These patients presented with typical nephrotic syndrome and PLA2R-related pMN with IgG4 predominance and very weak or absent IgG1 and C1q in the immune deposits. Therefore, a role for alternative complement pathway activation in pMN has also been suggested and warrants further investigation.

The complement system plays an important role in both innate and adaptive immunity. The classical pathway, the lectin pathway, and the alternative pathway all lead to cleavage of C3 and activation of the common terminal pathway, with release of anaphylatoxins C3a and C5a and generation of the membrane attack complex (MAC), which leads to sublytic injury of glomerular podocytes by compromising the cytoskeletal system, which plays an indispensable role in maintain the normal structure and function of podocytes, causing loss of cell-matrix adhesions and leak of protein into the Bowman capsule [6].

Although serum levels of complement proteins are usually normal in pMN, recent studies indicate that measurement of circulating complement activation products may be a more sensitive way to detect on-going complement activation [8, 9]. In the current study, we measured plasma and urine levels of various complement components and activation products in patients with pMN and correlated the levels with clinical and pathological parameters. Our goal was to better establish a role for complement activation in human pMN as well as to identify complement-related biomarkers of disease activity and explore potential therapeutic targets in pMN.

## Materials and methods

### Patients

One hundred thirty-four patients with biopsy-proven pMN, diagnosed in Peking University First Hospital from 2009 to 2013, were enrolled in our study. Patients with secondary MN were excluded including patients with systemic lupus erythematosus, hepatitis B virus infection, malignancy, medication and heavy metal poisoning related MN. Seventy-two patients with primary focal segmental glomerulosclerosis (FSGS) and 18 patients with minimal change disease (MCD) were enrolled as disease controls. Medical records and laboratory data were acquired at the time of kidney biopsy and during follow-up. 25 age- and gender-matched healthy donors were collected as healthy controls. They were all of negative urine test and normal serum creatinine.

Follow-up started at the time of renal biopsy and ended at either one of the following (whichever arrived first): 1) Dec 31, 2017; 2) the date of diagnosis of end-stage renal disease.

The study was performed in compliance with the Declaration of Helsinki and approved by the Ethics Committee of Peking University First Hospital. Written informed consent for obtaining tissue, blood and urine samples was obtained from each participant.

### Sample collection

All the plasma and urine samples of the patients with pMN, FSGS and MCD were collected on the day of kidney biopsy. Plasma samples were collected with disodium-EDTA as anticoagulant. All samples were stored frozen in aliquots at − 80 °C until measurements were made. Repeated freeze/thaw cycles were avoided.

Urine samples were available in 106 out of the 134 patients with pMN, in 56 out of the 72 patients with FSGS and in all the 18 patients with MCD. The clinical and pathological features were comparable between the patients with and without urine samples.

### Quantification of complement components levels

Commercial enzyme-linked immunosorbent assay (ELISA) kits were used to examining the level of human plasma/urinary complement components, including C4d, Bb, properdin, C3a, C5a and soluble C5b-9 (SC5b-9) (Quidel, San Diego, CA, USA), and C1q and MBL (BIOPRTO, Hellerup, Denmark) as described previously [10].

The level of plasma complement was corrected by eGFR (corrected level = measured level/eGFR) and the unit was (μg/ml)/(ml/min/1.73m^2^) for C1q, C4d, Bb, properdin, and (ng/ml)/(ml/min/1.73m^2^) for C3a, C5a, MAC, MBL. The level of urinary complement was corrected by urinary creatinine and urinary protein (corrected level = measured level/(urinary creatinine × urinary protein)) and the unit was ng/mg/g for C1q, MBL, Bb, MAC, and pg/mg/g for C3a, C5a, pg/mg/g properdin, and μg/mg/g for C4d.

### Detection of circulating anti-PLA_2_R antibodies

Circulating anti-PLA2R antibodies were detected using commercial ELISA kits (EUROIMMUN AG, Lübeck, Germany) following the protocols provided along with the kits. We first diluted the plasma samples with PBS containing 0.05% Tween-20 (PBST) at dilution ration of 1:100. The diluent was transferred to 96-well plates precoated with PLA2R antigen and then incubated at 25 °C for thirty minutes. The wells were washed for 4 times with washing buffer from the kits, and then co-incubated with enzyme-conjugated secondary antibodies at 25 °C for another thirty minutes. A microplate reader (Bio-Rad 550, Tokyo, Japan) was applied to detect and document the net optical absorbance at 450 nm.

Antibody positivity was defined as a level greater than 20 U/ml [11].

### Treatments and response

The use of corticosteroids and immunosuppressive agents, and the definitions of remission and relapse were in compliance with the 2012 KDIGO (Kidney Disease: Improving Global Outcomes) guideline for glomerulonephritis [12].

Estimated glomerular filtration rate (eGFR) was calculated using the Modification of Diet in Renal Disease (MDRD) Study equation adjusted for Chinese populations: eGFR = 175 × (plasma creatinine)-1.234 × age-0.179 × 0.79 (if female) [13].

To define the renal outcomes, the primary endpoint was end-stage renal disease (ESRD). If patients did not reach ESRD, the secondary endpoint was renal dysfunction, defined as eGFR decreased more than 30%, compared with baseline (at the time of renal biopsy) and the final eGFR being less than 60 ml/min/1.73m^2^.

### Statistical analysis

Student’s *t* test for normally distributed data, or nonparametric test (Mann–Whitney U test) for non-normally distributed data was used for the differences of quantitative parameters between groups. Correlations were analyzed by Pearson’s correlation test (between two normally distributed variables) or Spearman’s correlation test (between two non-normally distributed variables). Risk factors for no-remission in primary MN patients were analyzed using Logistic regression model. Results were expressed as odds ratio (OR) and 95% confidence interval (CI). Risk factors for renal outcomes were analyzed using a Cox regression model. Results were expressed as hazard ratios (HR) and 95% CI. All statistical analyses were two-tailed and differences of *P* < 0.05 were considered significant. Analysis was performed with SPSS statistical software package, version 19.0 (SPSS Inc., Chicago, IL, USA).

## Results

### Complement activation in the plasma and urine of patients with pMN

The demographic, clinical and pathological characteristics of the 134 patients with pMN were shown in Table 1, and were compared to those with FSGS and MCD and healthy controls.

**Table 1:** The demographic, clinical and pathological features of the patients with pMN, FSGS and MCD

Parameters	PMN (*N* = 134)	FSGS (*N* = 72)	MCD (*N* = 18)	*P*
Gender (male), n (%)	79 (59.0)	48 (66.7)	8 (44.4)	0.201
Age (years)	52.0 (18.0–76.0)	28.5 (13.0–84.0)	55.5 (15.0–81.0)	< 0.001
Nephrotic syndrome	79 (59.0%)			
Albumin (g/L)	26.4 (15.1–44.7)	19.9 (12.0–43.9)	24.0 (14.5–39.4)	< 0.001
Serum creatinine (μmol/L)	63.1 (33.4–119.6)	94.0 (43.0–931.0)	63.3 (42.5–164.2)	< 0.001
eGFR (ml/min/1.73m^2^)	120.7 (56.6–248.5)	82.0 (4.7–281.6)	108.7 (30.4–266.1)	< 0.001
Proteinuria (g/24 h), median (range)	4.3 (0.2–20.9)	7.6 (0.8–27.9)	3.8 (0.02–18.2)	< 0.001
Anti-PLA2R antibody positivity, n (%)	91 (67.9%)	–	–	–
Anti-PLA2R antibody levels (+) (RU/ml)	83.9 (22.5–384.9)	–	–	–
Anti-PLA2R antibody levels (RU/ml)	38.4 (0.0–384.9)	–	–	–
IgG staining, n (%)	130 (97.0)	1 (1.4)	3 (16.7)	< 0.001
IgA staining, n (%)	27 (20.2)	6 (8.3)	1 (5.6)	0.045
IgM staining, n (%)	42 (31.3)	42 (58.3)	9 (50.0)	0.001
C3-staining, n (%)	116 (86.6)	14 (19.4)	5 (27.8)	< 0.001
C1q-staining, n (%)	31 (23.1)	3 (4.2)	0 (0.0)	< 0.001
Stage I glomerular lesion, n (%)	59 (44.0%)	–	–	–
Stage II glomerular lesion, n (%)	61 (45.5%)	–	–	–
Stage III glomerular lesion, n (%)	14 (10.5%)	–	–	–

Data was shown as median (range)

**Fig. 1:**
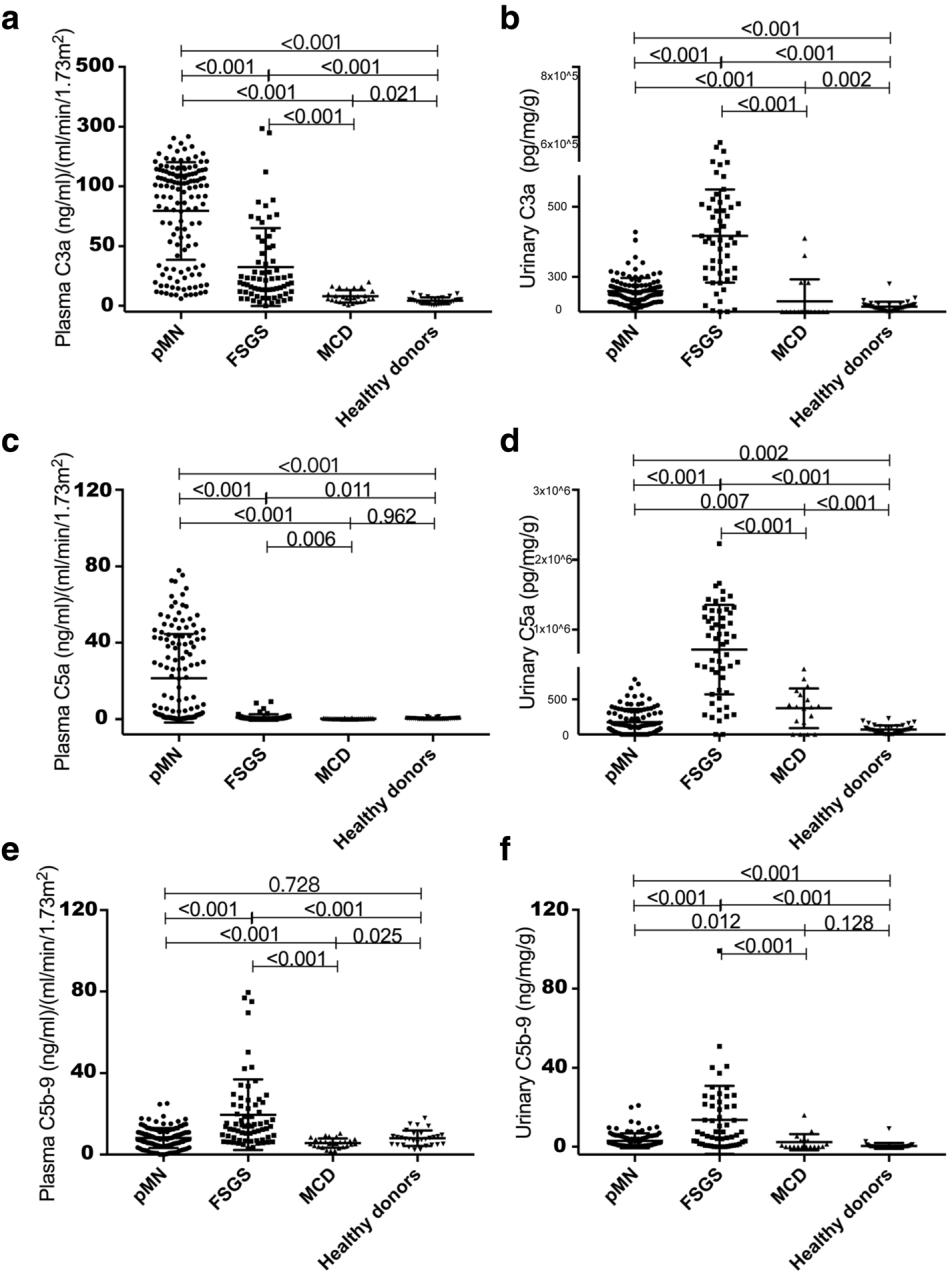
Complement activation in the plasma and urine of the patients with pMN, FSGS, and MCD and healthy controls. The plasma values were corrected by eGFR and the urine values were corrected by urinary creatinine and urinary protein. All the data were log_10_-transformed

All the plasma levels of complement components were corrected by eGFR of each patient to exclude the possibility that the elevated values reflect lower eGFR. All the urinary levels of complement components were corrected by urinary creatinine and urinary protein to exclude the possibility that the elevated values reflect lower glomerular filtration or higher proteinuria (Fig. 1a-f, Table 2).

**Table 2:** The comparisons of complement component in the pMN, FSGS, MCD patients and healthy controls

	Value, median (IQR)	Abnormal, n (%)	Value, median (IQR)	Abnormal, n (%)	Value, median (IQR)	Abnormal, n (%)	Value, median (IQR)	Cut-off value
	pMN (*n* = 134)		FSGS (*n* = 72)		MCD (*n* = 18)		Healthy Controls (*n* = 40)	
P-C1q	0.8 (0.5–1.1)	24 (17.9)	0.9 (0.6–1.8) **	25 (36.8) **	0.6 (0.4–0.7) *	1 (3.6)	0.6 (0.5–0.9)	1.2
P-C4d	0.2 (0.1–0.3)	134 (100)	0.2 (0.1–0.3)	67 (98.5)	0.01 (0.00–0.01) **	0 (0) **	0.02 (0.01–0.02) **	0.03
P-MBL	19.5 (6.8–35.9)	24 (17.9)	1.1 (0.5–2.7) **	0 (0) **	23.9 (8.7–37.9)	5 (17.9)	16.3 (6.8–23.8)	41.8
P-Bb	0.008 (0.005–0.01)	28 (20.9)	0.01 (0.00–0.02)	17 (25.0)	0.005 (0.004–0.008) **	3 (10.7)	0.006 (0.004–0.008) **	0.01
P-Properdin	0.2 (0.1–0.3)	2 (1.5)	–	–	0.2 (0.1–0.3)	0 (0)	0.2 (0.1–0.3)	0.5
P-C3a	85.8 (11.8–120.0)	128 (95.5)	6.4 (3.2–20.8) **	54 (79.4) **	1.7 (1.0–3.3) **	7 (25.0) **	0.9 (0.4–1.5) **	2.5
P-C5a	2.4 (0.3–23.8)	100 (74.6)	0.1 (0.1–0.3) **	14 (20.6) **	0.05 (0.02–0.11) **	0 (0) **	0.6 (0.03–0.11) **	0.3
P-MAC	2.4 (1.4–3.8)	8 (6.0)	4.9 (2.8–10.3) **	28 (41.2) **	1.7 (1.2–2.3) **	0 (0)	2.5 (1.5–3.3)	5.6
	pMN (*n* = 106)		FSGS (*n* = 55)		MCD (n = 18)		Healthy Controls (n = 40)	
U-C1q	0.0 (0.0–0.2)	6 (5.7)	0.3 (0.1–0.7) **	2 (3.6)	0.0 (0.0–0.3)	2 (11.1)	1.1 (0.0–2.8) **	5.9
U-C4d	0.1 (0.0–1.2)	37 (34.9)	–	–	13.0 (5.5–25.0) **	17 (94.4) **	0.0 (0.0–0.7) **	0.7
U-MBL	0.0 (0.0–0.0)	10 (9.4)	0.8 (0.0–5.7) **	47 (85.5) **	0.0 (0.0–0.0)	0 (0)	0.0 (0.0–0.0)	0.0
U-Bb	1.0 (0.4–1.9)	2 (1.9)	0.01 (0.01–0.10) **	0 (0)	10.5 (5.6–28.2) **	8 (44.4) **	4.5 (1.5–4.6) **	10.8
U-Properdin	0.0 (0.0–13.7)	16 (15.1)	–	–	654.3 (213.9–1775.7) **	18 (100) **	0.0 (0.0–0.0) *	28.9
U-C3a	21.5 (10.8–46.5)	53 (50)	1257.9 (118.3–12,195.0) **	48 (87.3) **	0.0 (0.0–14.7) **	4 (22.2) *	3.7 (1.6–5.8) **	22.2
U-C5a	7.9 (0.7–33.6)	40 (37.7)	4398.6 (173.4–29,984.6) **	53 (96.4) **	51.1 (9.2–137.1) *	13 (72.2) *	3.3 (1.5–6.5) *	13.3
U-MAC	1.0 (0.3–2.6)	31 (29.2)	5.4 (0.8–24.7) **	36 (65.5) **	0.0 (0.0–2.14) *	4 (22.2)	0.0 (0.0–0.01) **	2.5

*P* plasma, *U* urine. *: *P* < 0.05; **: *P* < 0.001; compared to pMN patients. Data was shown as median (inter-quartile range). Cut-off value was defined as mean + 2 SD. The level of plasma complement was corrected by eGFR (corrected level = measured level/eGFR) and the unit was and (μg/ml)/(ml/min/1.73m^2^) for C1q, C4d, Bb, properdin, and (ng/ml)/(ml/min/1.73m^2^) for C3a, C5a, MAC, MBL. The level of urinary complement was corrected by urinary creatinine and urinary protein (corrected level = measured level / (urinary creatinine × urinary protein)) and the unit was ng/mg/g for C1q, MBL, Bb, MAC, and pg/mg/g for C3a, C5a, pg/mg/g properdin, and μg/mg/g for C4d

The plasma levels of C3a were significantly higher in patients with pMN than those in FSGS (*P* < 0.001), MCD (*P* < 0.001) and healthy controls (*P* < 0.001). The urinary levels of C3a in pMN were significantly lower than those in FSGS (*P* < 0.001), higher than those in MCD (*P* < 0.001) and normal controls (*P* < 0.001).

The plasma levels of C5a in patients with pMN were significantly higher than those in FSGS (*P* < 0.001), MCD (*P* < 0.001) and healthy controls (*P* < 0.001). The urinary levels of C5a in pMN were significantly lower than those in FSGS (*P* < 0.001) and MCD (*P* = 0.007), and higher than healthy controls (*P* = 0.001).

The plasma levels of C5b-9 in patients with pMN were significantly lower than those in FSGS (*P* < 0.001), higher than MCD (P < 0.001), and comparable with healthy controls (*P* = 0.728). The urinary levels of C5b-9 in patients with pMN were significantly lower than those in FSGS (*P* < 0.001), higher than MCD (*P* = 0.012) and healthy controls (*P* < 0.001).

### The pathways of complement activation

**Fig. 2:**
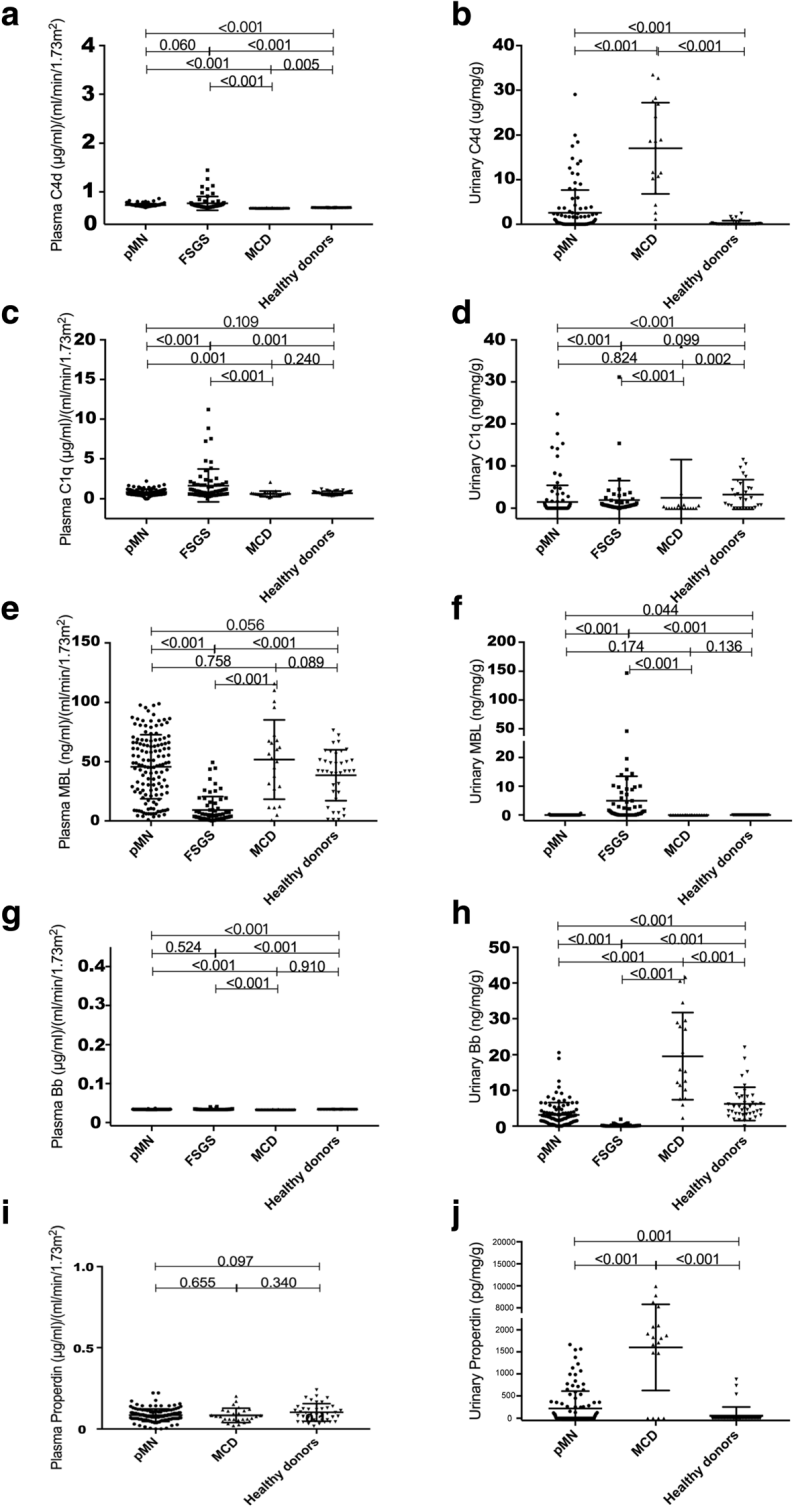
The pathways of complement activation in the plasma and urine of the patients with pMN, FSGS, and MCD and healthy controls. The plasma values were corrected by eGFR and the urine values were corrected by urinary creatinine and urinary protein. All the data were log_10_-transformed

The components of classical pathway, lectin pathway, and alternative pathway of complement activation were assessed in the patients with pMN, and compared to those in the patients with FSGS and MCD and healthy controls (Fig. 2a-j, Table 2).

The plasma levels of C4d in patients with pMN were comparable to those in FSGS (*P* = 0.060), higher than MCD (*P* < 0.001) and healthy donor (*P* < 0.001). The urinary levels of C4d in patients with pMN were higher than healthy controls (*P* < 0.001), and lower than those in MCD (*P* < 0.001).

The plasma levels of C1q in patients with pMN were lower than those in FSGS (*P* < 0.001), higher than MCD (*P* = 0.001), and comparable with healthy controls (*P* = 0.109). The urinary levels of C1q in patients with pMN were lower than those in FSGS (*P* < 0.001) and healthy controls (*P* < 0.001), but comparable to those in MCD (*P* = 0.836).

The plasma levels of MBL in patients with pMN were higher than those in FSGS (*P* < 0.001), and comparable to MCD (*P* = 0.758) and healthy controls (*P* = 0.056). The urinary levels of MBL in patients with pMN were lower than those in FSGS (*P* < 0.001) and higher than healthy controls (*P* = 0.044), but comparable to those in MCD (*P* = 0.174).

The plasma levels of Bb in patients with pMN were comparable to those in FSGS (*P* = 0.524), and higher than MCD (*P* < 0.001) and healthy controls (*P* < 0.001). The urinary levels of Bb in patients with pMN were higher than those in FSGS (*P* < 0.001), and lower than those in MCD (*P* < 0.001) and health controls (*P* < 0.001). The plasma levels of properdin in patients with pMN were comparable to those in MCD (*P* = 0.655) and healthy donors (*P* = 0.097). The levels of urinary properdin in patients with pMN were lower than those in MCD (*P* < 0.001), and higher than healthy controls (*P* = 0.001).

### Association between the levels of complement components and clinical data

Ninety-one (67.9%) out of the 134 patients with pMN were positive for anti-PLA2R antibodies, with an average level of 83.9 (50.1, 190.5) U/ml. Between the patients with positive or negative anti-PLA2R antibodies, no difference was shown on the serum or urinary levels of complement components (Table 3).

**Table 3:** The comparisons of complement component in the pMN patients with or without anti-PLA2R antibodies

	PLA2R-Ab (+) (*n* = 91)	PLA2R-Ab (−) (*n* = 43)	Healthy controls
P-C1q	0.8 (0.6–1.1)	0.7 (0.5–0.9)	0.7 (0.5–0.9)
P-C4d	0.2 (0.2–0.3) **	0.2 (0.1–0.2) **	0.02 (0.01–0.02)
P-MBL	19.8 (6.7–38.3)	19.5 (2.5–32.7)	16.3 (6.8–23.8)
P-Bb	0.01 (0.01–0.01) **	0.01 (0.00–0.01) **	0.01 (0.00–0.01)
P-Properdin	0.2 (0.2–0.3)	0.2 (0.1–0.2)	0.2 (0.1–0.3)
P-C3a	79.5 (11.8–118) **	88.1 (10.3–127.8) **	0.9 (0.4–1.5)
P-C5a	3.7 (0.3–23.9) **	1.8 (0.3–23.8) **	0.06 (0.03–0.11)
P-MAC	2.6 (1.6–3.7)	2.3 (1.2–4.0)	2.5 (1.5–3.3)
U-C1q	0.00 (0.00–0.02) **	0.00 (0.00–0.23) **	1.11 (0.04–2.8)
U-C4d	0.05 (0.00–0.89) *	0.02 (0.00–0.019)	0.00 (0.00–0.07)
U-MBL	0.00 (0.00–0.00)	0.0 (0.0–0.0)	0.00 (0.00–0.00)
U-Bb	0.7 (0.0–1.6) **	0.9 (0.2–1.8) **	2.5 (1.7–4.6)
U-Properdin	0.0 (0.0–7.9) *	0.0 (0.0–2.2)	0.0 (0.0–0.0)
U-C3a	12.5 (2.4–36.7) **	21.1 (8.6–42.7) **	3.7 (1.6–5.8)
U-C5a	4.9 (0.0–30.3) *	1.5 (0.0–11.0)	3.3 (1.5–6.5)
U-MAC	0.6 (0.0–2.0) **	0.38 (0.05–3.26) **	0.0 (0.0–0.1)

*P* plasma, *U* urine. *: *P* < 0.05; **: *P* < 0.01; compared to healthy controls. Data was shown as median (inter-quartile range). No difference was found between the patients with or without anti-PLA2R antibodies (*P* > 0.05)

**Fig. 3:**
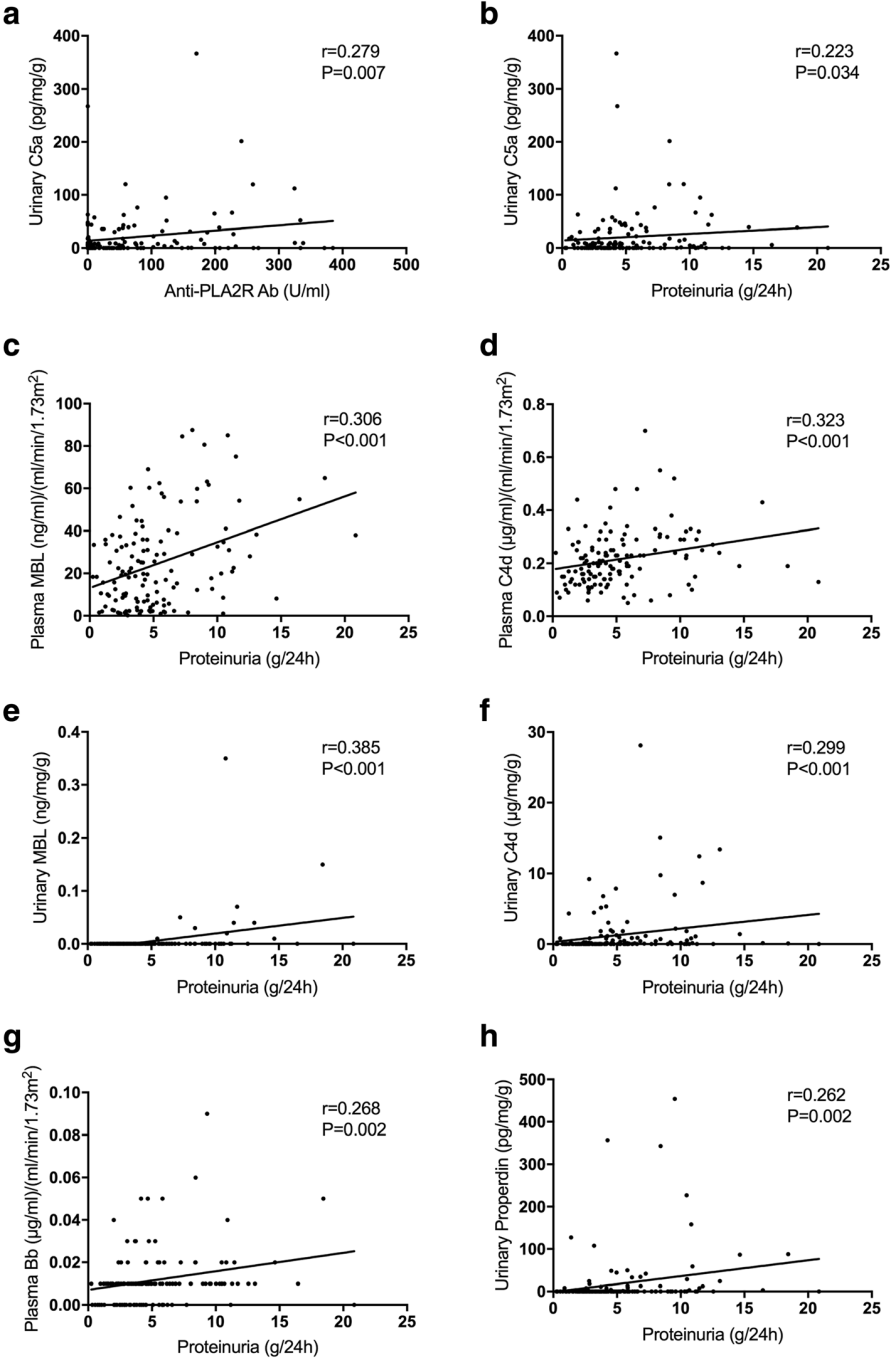
Correlations between the levels of complement components and the clinical data of patients with pMN. The plasma values were corrected by eGFR and the urine values were corrected by urinary creatinine and protein excretion. The analyses were performed using Spearman’s rank correlation

The urinary levels of C5a were positively correlated with the levels of anti-PLA2R antibodies (*r* = 0.279, *P* = 0.007) and the urinary protein (*r* = 0.223, *P* = 0.034) (Fig. 3a, b) in the patients with positive anti-PLA2R antibodies. No other complement component showed any association with the positivity or the levels of anti-PLA2R antibodies.

The circulating MBL levels were positively correlated with the urinary protein excretion (*r* = 0.306, *P* < 0.001) (Fig. 3c). This correlation was shown in the PLA2R antibody positive patients (*r* = 0.307, *P* = 0.003), but not in the patients with negative antibody (*P* > 0.05). Similar phenomenon was observed for plasma C4d. The circulating C4d levels were positively correlated with the urinary protein excretion (*r* = 0.323, *P* < 0.001) (Fig. 3d). This correlation was shown in the PLA2R antibody positive patients (*r* = 0.379, *P* < 0.001), but not in the patients with negative antibody (*P* > 0.05). The urinary levels of MBL and C4d showed positive correlation with the urinary protein (MBL: *r* = 0.385, *P* < 0.001; C4d: *r* = 0.299, *P* < 0.001) (Fig. 3e, f), both in the patients with anti-PLA2R antibodies (MBL: *r* = 0.384, *P* < 0.001; C4d: *r* = 0.270, *P* = 0.010) and those without (MBL: *r* = 0.365, *P* = 0.016; C4d: *r* = 0.404, *P* = 0.007).

The circulating Bb levels were positively correlated with the urinary protein excretion (*r* = 0.268, *P* = 0.002) (Fig. 3g). This correlation was shown in the PLA2R antibody negative patients (*r* = 0.386, *P* = 0.010), but not in the patients with positive antibody (*P* > 0.05). The urinary levels of properdin showed positive correlation with the urinary protein (*r* = 0.262, *P* = 0.002) (Fig. 3h). This correlation was observed only in patients with negative antibody (*r* = 0.368, *P* = 0.015), but not in the patients with positive anti-PLA2R antibodies (*P* > 0.05).

**Fig. 4:**
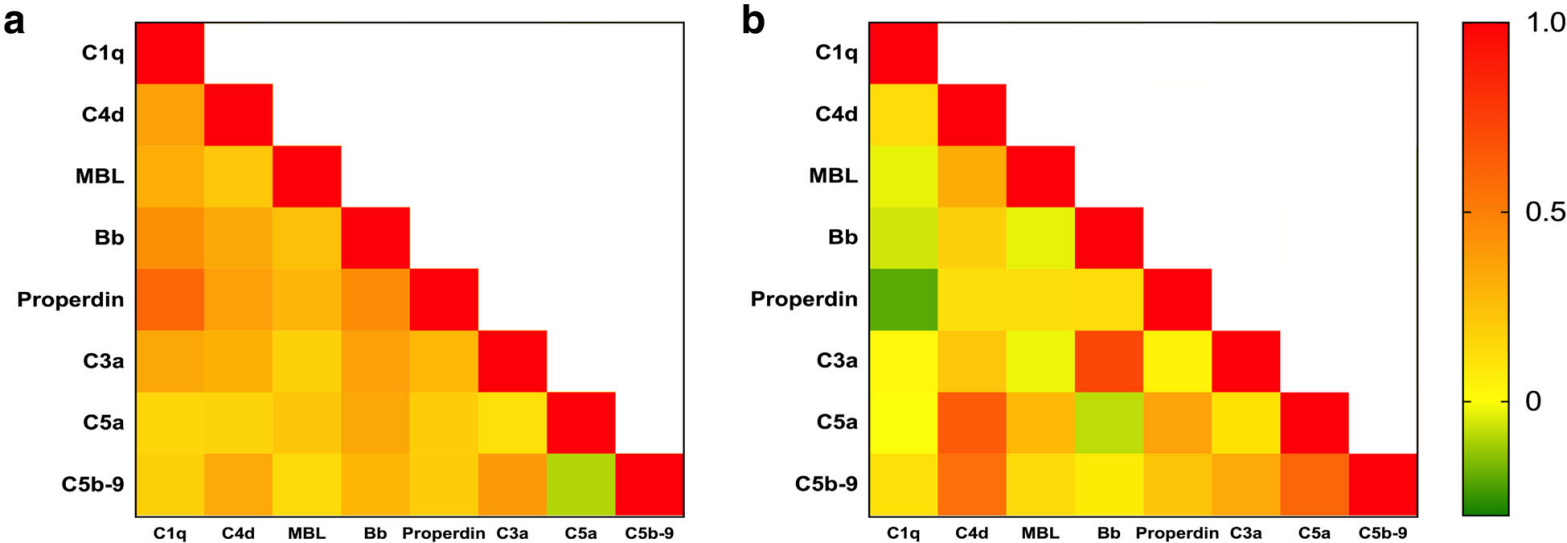
The heatmap of inter-correlations among complement components in pMN patients. **a**: plasma levels. **b**: urinary levels. **a** and **b** indicated that there were strong correlation between the complement components

There were inter-correlations among the complements in plasma or those in urine of the patients with pMN (Fig. 4a, b). No correlation was observed between the plasma levels of any complements and their urinary levels (*P* > 0.05).

### Association between the complement levels and treatment responses and kidney outcomes

118/134 (86.8%) patients were followed for 33.8 ± 17.2 months. Among them, 90 (76.3%) patients achieved remission, including 51 (43.2%) patients with partial remissions and 39 (33.1%) with complete remissions. Twenty-six patients of 118 (22.0%) suffered from kidney dysfunction (eGFR decreased more than 50% compared to baseline on kidney biopsy) and two patients developed ESRD.

Using Logistic regression analysis, we found that the higher level of anti-PLA2R antibodies, the lower level of serum albumin, and the lower level of serum C3 were risk factors of no-remission. However, multivariate analysis showed that none of the predictors were significance (Table 4).

**Table 4:** Logistic regression analysis of the risk factors for no-remission in the patients with pMN

Parameters	Univariate analysis		Multivariate analysis	
	OR (95% CI)	*P* value	OR (95% CI)	*P* value
Gender (male)	0.927 (0.390, 2.205)	0.864		
Age	0.999 (0.968, 1.031)	0.968		
Nephrotic syndrome	0.857 (0.364, 2.016)	0.724		
Anti-PLA2R antibody positivity	0.375 (0.130, 1.082)	0.070		
Level of anti-PLA2R antibodies	1.005 (1.001, 1.009)	**0.016**	0.999 (0.992–1.006)	0.773
Proteinuria	1.117 (0.997, 1.251)	0.056		
Serum albumin	0.918 (0.847, 0.996)	**0.040**	0.893 (0.793–1.005)	0.059
eGFR	0.998 (0.987, 1.010)	0.762		
Serum-C3	0.092 (0.010, 0.880)	**0.038**	0.967 (0.045–20.88)	0.983
Serum-C4	0.195 (0.000, 271.8)	0.658		
P-C1q	2.848 (0.710–11.422)	0.140		
P-C4d	5.651 (0.046–687.1)	0.480		
P-MBL	1.016 (0.992–1.040)	0.191		
P-Bb	382.258 (0.0–1351.0)	0.760		
P-Properdin	22.398 (0.071–7064)	0.290		
P-C3a	1.001 (0.992–1.010)	0.870		
P-C5a	1.013 (0.987–1.040)	0.330		
P-MAC	0.671 (0.449–1.005)	0.053		
U-C1q	1.063 (0.878–1.286)	0.530		
U-C4d	1.086 (0.973–1.213)	0.141		
U-MBL	1.700 (0.0–8,369,066.8)	0.946		
U-Bb	0.890 (0.617–1.283)	0.533		
U-Properdin	1.001 (0.993–1.009)	0.824		
U-C3a	1.000 (0.998–1.003)	0.731		
U-C5a	1.003 (0.994–1.012)	0.530		
U-MAC	1.063 (0.938–1.206)	0.338		

*P* plasma, *U* urine

The *p* values with statistical significance were showed in bold (*P* < 0.05)

Cox regression analysis showed that male gender and higher level of anti-PLA2R antibodies were risk factors for kidney dysfunction during follow-up. Multivariate analysis showed that the higher level of anti-PLA2R antibodies (HR = 1.005, 95% CI 1.001–1.01, *P* = 0.023) was the only risk factor to kidney dysfunction (Table 5).

**Table 5:** Cox regression analysis of the risk factors for renal dysfunction in the patients with pMN

Parameters	Univariate analysis		Multivariate analysis	
	HR (95% CI)	*P* value	HR (95% CI)	*P* value
Gender (male)	2.403 (1.089–5.305)	**0.030**	1.500 (0.448–5.015)	0.511
Age	1.031 (0.980–1.086)	0.238		
Nephrotic syndrome	0.741 (0.216–2.543)	0.634		
Anti-PLA2R antibody positivity	0.249 (0.032–1.945)	0.185		
Level of anti-PLA2R antibodies	1.006 (1.001–1.010)	**0.020**	1.005 (1.001–1.010)	**0.023**
Proteinuria	1.031 (0.879–1.209)	0.712		
Serum albumin	0.983 (0.882–1.075)	0.750		
eGFR	1.005 (0.988–1.023)	0.566		
Serum-C3	0.242 (0.013–4.413)	0.338		
Serum-C4	0.011 (0.00–92.092)	0.329		
P-C1q	0. 721 (0.120–4.321)	0.721		
P-C4d	0.213 (0.003–17.880)	0.494		
P-MBL	0.983 (0.962–1.004)	0.119		
P-Bb	0.000 (0.000–5820)	0.381		
P-Properdin	0.090 (0.000–67.712)	0.476		
P-C3a	0.990 (0.978–1.002)	0.101		
P-C5a	0.989 (0.955–1.024)	0.554		
P-MAC	0.924 (0.689–1.240)	0.600		
U-C1q	0.642 (0.100–4.107)	0.640		
U-C4d	1.026 (0.932–1.129)	0.607		
U-MBL	0.739 (0.000–5,302,470)	0.970		
U-Bb	1.020 (0.85–1.216)	0.828		
U-Properdin	0.978 (0.922–1.037)	0.459		
U-C3a	1.000 (0.998–1.022)	0.856		
U-C5a	1.003 (0.995–1.011)	0.442		
U-MAC	0.991 (0.859–1.144)	0.905		

*P* plasma, *U* urine

The *p* values with statistical significance were showed in bold (*P* < 0.05)

No complement component showed effect to predict the treatment responses or kidney outcomes of patients with pMN.

## Discussion

The immunopathology of human pMN consistently shows evidence of complement activation within immune deposits [14]. Sublytic injury of podocytes due to membrane insertion of MAC has been well established to mediate proteinuria in the Heymann nephritis animal models of MN [15]. Based on these observations it is thought likely that the complement system also plays a substantial role in the pathogenesis of the human disease. In the current study, we demonstrate significant elevated serum and urinary levels of complement activation products in the patients with pMN, which may be a more sensitive measurement of the on-going complement activation in the kidney. All the plasma levels of complement components were corrected by eGFR to exclude the possibility that the elevated values reflect lower eGFR. All the urinary levels of complement components were corrected by urinary creatinine and urinary protein excretion to exclude the possibility that the elevated values reflect lower glomerular filtration or higher proteinuria.

We found remarkable increments of C3a and C5a in the circulation of patients with pMN. Compared to the patients with FSGS and MCD, whom presented characteristically with nephrotic syndrome as well, the patients with pMN showed several to tens fold higher levels of serum C3a and C5a. C3a level was even much higher than C5a level. C3aR and C5aR are widely distributed and are expressed on most hematopoietic cells, including neutrophils, monocytes/macrophages, basophils/mast cells, T cells, B cells and dendritic cells [16–19]. However, in the kidney intrinsic cells, C3aR but not C5aR is expressed on the membrane of podocyte [20–22]. C3a is of low molecule weight (9kD) and can pass through the glomerular filtration barrier freely, exerting an anaphylatoxin effect by binding to G-protein-coupled receptors [23]. C3a appears to have key roles in inflammatory disorders such as asthma and ischemia-reperfusion injury in the kidney [24]. Chronic administration of C3aR antagonist to MRL/lpr lupus mice significantly reduced kidney disease and prolonged survival [20]. We suspect that C3aR signaling might also participate in the complement-mediated dedifferentiation of podocyte phenotype in pMN. However, C3a/C3aR have not yet been studied in pMN.

The serum C5a level was significantly higher in the patients with pMN, and a positive correlation was observed between the urinary C5a levels and the anti-PLA2R antibody levels. One possible explanation is that the interaction between anti-PLA2R antibodies and podocytes membrane antigen in the subepithelial space results in local activation of C3. Thus, urine C5a would increase as the anti-PLA2R antibody level increases and more deposits are being formed. The elevated serum C3a and C5a level also implies a possible interaction between complement activation and PLA2R-specific adaptive autoimmune disorder. Previous studies have revealed the relationship between complement components and adaptive immune response, in which deficiency of complement components decreases quantity of antibodies. The mechanism of this bond still remains unclear, and a possible explanation is that iC3b cleaves into C3dg, which binds with antigen in circulation and the antigen-C3dg complex binds to CR2 (CD21) expressed on B cells. The antigen presentation process was facilitated with the presence of C3dg, thus lowering the threshold for B cell activation [25–27].

The roles of complement in modulating the adaptive PLA2R immune response need further investigations. Although one trial of the C5 inhibitor eculizumab was negative in pMN [9, 28], adequate complement-inhibiting doses were not used. Such high levels of srum C3a and C5a indicate that the inhibitors against C3a/C3aR or C5a/C5aR still may be reasonable for trials, or the therapies target to complement should be applied to the patients with high level of complement activation.

In this study, MAC (sC5b-9) was increased only in the urine of patients with pMN but remained in normal range in the circulation. As well, no correlation was found between plasma and urine levels of any complement components. This indicates that MAC formation is occurring predominantly in situ in the kidney. C5b-9 inserted into the podocytes can be transported intracellularly and extruded into the urinary space, where it subsequently appears in the urine [29]. Insertion of MAC in podocytes is a sublethal event, and the cell membrane repairs rapidly [30]. Thus, the urinary C5b-9 is a dynamic marker of ongoing immunological injury.

This study observed a general activation of classical pathway, lectin pathway, and alternative pathway in patients with pMN. However, different interpretations were proposed after further analysis, serving in the common pathway activation and to the clinic features. 1. The urinary C1q level was lower than and the serum C1q was within normal range. No correlation was identified between the C1q levels and any clinical features. C1q deposition is typically absent in the kidney specimens of patients with pMN [7, 9]. Thus, although C1q might be activated at a low level by the detectable but usually low levels of IgG1 and/or IgG3 subclasses of anti-PLA2R antibodies [14], the classical pathway is probably not a major player in the pathogenesis of pMN. 2. The levels of circulating and urinary C4d was elevated, while the serum and urinary MBL were comparable to healthy controls. These findings imply that in the lectin pathway of complement activation in pMN, there might be other participants other than MBL of activating MASP and C4 [31]. 3. Positive correlations to proteinuria was shown with MBL and C4d only in the patients with positive anti-PLA2R antibodies but not in the patients with negative antibody. MBL has been identified in the glomeruli of patients with pMN [7]. Preliminary studies report that affinity purified IgG4 anti-PLA2R antibodies could bind MBL and promote C4 deposition [32]. MBL has been shown to activate complement in patients with rheumatoid arthritis by binding to IgG Fc that is deficient in terminal galactose, thus exposing GlcNAc [33]. Further studies are needed to explore whether a similar mechanism is at work in the case of IgG4 anti-PLA2R antibodies. 4. Positive correlations between factor B/properdin and urinary protein were observed only in the patients with negative antibody but not in the patients with positive anti-PLA2R antibodies. Deposits of factor B suggest activation of the alternative pathway in the kidney in situ [34]. The cases in which pMN developed with complete MBL deficiency are consistent with an independent role for the alternative pathway in complement activation and kidney injury in some patients with pMN [7]. The current findings indicate that the alternative pathway of complement activation of pMN may occur in the absence of autoantibody. This may be one explanation for the high prevalence of C3 deposits in pMN patients either with or without antibody. The motivator for the alternative pathway activation needs further investigations.

There were several limitations of our study, including a relatively small sample size, especially the number of controls, and limited length of follow-up. Multi-center analysis with larger sample size and longer follow-up should be performed in the future.

## Conclusions

In conclusion, this study provides the changing of complement activation products in the serum and urine of patients with pMN. The remarkable increments of C3a and C5a indicate strong activation of complement and correlation with anti-PLA2R antibody levels. The complement may be activated through lectin pathway in the patients with positive anti-PLA2R antibodies, and through alternative pathway in the patients with negative antibody. These complement-related mechanisms may provide potential therapeutic targets in patients with pMN.

The highlight of this study is that we explored all three pathways of complement activation and correlate anti-PLA2R antibody with complement for the first time. The relatively small sample size, however, is a major limitation of this study, which requires further expansion of the cohort and multicenter investigations.

## Data Availability

The datasets used and analyzed during the current study are available from the corresponding author on reasonable request.

## Abbreviations

APC: Antigen-presenting cell
ELISA: Enzyme-linked immunosorbent assay
ESRD: End-stage renal disease
FSGS: Focal segmental glomerulosclerosis
HR: Hazard ratios
MAC: Membrane attack complex
MCD: Minimal change disease
PLA2R: M-type phospholipase A2 receptor
pMN: primary membranous nephropathy
